# Respiratory viral infections in the elderly

**DOI:** 10.1177/1753466621995050

**Published:** 2021-03-21

**Authors:** Alastair Watson, Tom M. A. Wilkinson

**Affiliations:** Faculty of Medicine, Clinical & Experimental Sciences, University of Southampton, Southampton, UK; NIHR Southampton Biomedical Research Centre, University Hospital Southampton NHS Foundation Trust, Southampton, UK; Birmingham Medical School, University of Birmingham, Birmingham, UK; Faculty of Medicine, Clinical and Experimental Sciences, Southampton University, Mailpoint 810, Level F, South Block, Southampton General Hospital, Southampton, Hampshire, SO16 6YD, UK; NIHR Southampton Biomedical Research Centre, University Hospital Southampton NHS Foundation Trust, Southampton, UK

**Keywords:** elderly, respiratory viruses, SARS-CoV-2, influenza, RSV, COVID-19, screening, therapeutics, vaccination, polypharmacy

## Abstract

With the global over 60-year-old population predicted to more than double over the next 35 years, caring for this aging population has become a major global healthcare challenge. In 2016 there were over 1 million deaths in >70 year olds due to lower respiratory tract infections; 13–31% of these have been reported to be caused by viruses. Since then, there has been a global COVID-19 pandemic, which has caused over 2.3 million deaths so far; increased age has been shown to be the biggest risk factor for morbidity and mortality. Thus, the burden of respiratory viral infections in the elderly is becoming an increasing unmet clinical need. Particular challenges are faced due to the interplay of a variety of factors including complex multimorbidities, decreased physiological reserve and an aging immune system. Moreover, their atypical presentation of symptoms may lead to delayed necessary care, prescription of additional drugs and prolonged hospital stay. This leads to morbidity and mortality and further nosocomial spread. Clinicians currently have limited access to sensitive detection methods. Furthermore, a lack of effective antiviral treatments means there is little incentive to diagnose and record specific non-COVID-19 viral infections. To meet this unmet clinical need, it is first essential to fully understand the burden of respiratory viruses in the elderly. Doing this through prospective screening research studies for all respiratory viruses will help guide preventative policies and clinical trials for emerging therapeutics. The implementation of multiplex point-of-care diagnostics as a mainstay in all healthcare settings will be essential to understand the burden of respiratory viruses, diagnose patients and monitor outbreaks. The further development of novel targeted vaccinations as well as anti-viral therapeutics and new ways to augment the aging immune system is now also essential.

*The reviews of this paper are available via the supplemental material section.*

## Introduction

With the United Nations (UN) predicting a doubling of the number of over 60-year-olds worldwide from 901 million in 2015 to 2.1 billion in 2050,^1^ ensuring overall quality of life in our aging population is now a major global public health challenge. Problems arise not only in dealing with an aging immune system and complex multimorbidities, but also in knowing how to identify, distinguish, prevent and treat the infectious diseases that often exacerbate morbidity in the elderly.

The burden of respiratory viral infections in the elderly has been highlighted by the severe acute respiratory syndrome coronavirus 2 (SARS-CoV-2) which has infected millions of people worldwide and had unprecedented impacts on healthcare systems and society as a whole.^2–4^ Age has been found to be the biggest risk factor for the development of severe COVID-19. Furthermore, a large United Kingdom (UK) prospective sobservation study demonstrated that COVID-19 mortality was over 11 times higher in >80-year-olds than in <50-year-olds.^3^

Other respiratory viruses, including influenza and respiratory syncytial virus (RSV) however also lead to substantial morbidity and mortality, particularly in the elderly.^5^ Similarly, there is an increasing understanding of the burden of human metapneumovirus (HMPV), human rhinovirus (HRV) and human parainfluenza virus (HPIV). Respiratory viral infections inhibit the ability of the elderly to function and carry out their everyday life tasks. Thus, having a substantial impact on the elderly individuals and their families. Furthermore, respiratory viruses are key contributors to exacerbation of chronic diseases.^6^

## Epidemiology

### SARS-CoV-2

Since the end of 2019, SARS-CoV-2 infection has caused 107.5 million cases of COVID-19 and >2.3 million deaths worldwide.^2^ A large prospective observational study in the UK found that 72.7% of those presenting to hospital with COVID-19 were >60 years old, highlighting the particular vulnerability of older adults to COVD-19. Numbers of deaths were also shown to increase with increasing age, with 12.8%, 29.2% and 50.4% of deaths seen in 60–69, 70–79 and 80+ year olds, respectively.^3^ This was mirrored in the United States (US) where 80% of COVID-19 disease was in the >65 years old population.^7^

**Figure 1.**
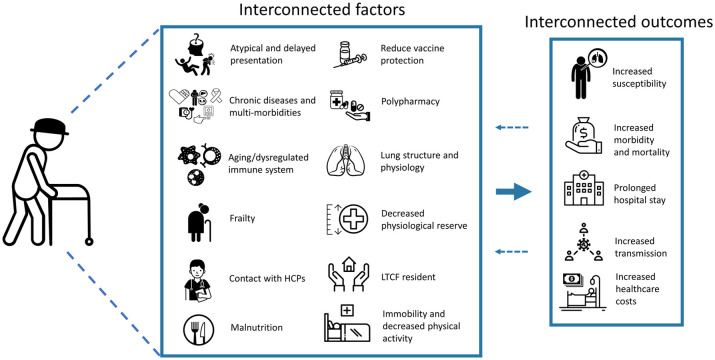
Interconnected factors that drive an increased susceptibility and impact of respiratory viral infections in the elderly. Figure was formed from images taken from The Noun Project.^159^ HCP, healthcare professional; LTCF, long-term-care facility. The Noun Project, 8800 Venice Blvd., Los Angeles, CA 90034. Work is licensed under the Attribution 3.0 Unported (CC BY 3.0) licence^©^. Images used were downloaded from https://thenounproject.com on 8 December 2020, and from top left to bottom right were produced by: Confusion by Mohammed Rabiul Alam, BD; falling by Andrew Doane; heart attack by Gan Khoon Lay; heart disease by artworkbean, ID; liver and kidney problem by Gan Khoon Lay; cancer by LAFS, RU; Blood Pressure Cuff by Shiva, IN; Diabetes by Daniel Grohotolski, DE; macrophage by Léa Lortal; natural killer cell by Léa Lortal; neutrophil by Léa Lortal; grandmother by Marie Van den Broeck, BE; Nurse by Llisole; Food by Guilherme Furtado, BR; Vaccine by parkjisun; medicine by UNiCORN; medicine by alvianwijaya, ID; lungs by Saeful Muslim, ID health by StringLabs, ID; home care by Bestdesignmarket, IN; Hospital Bed by Linseed Studio, US; Pneumonia by Gan Khoon Lay; cost by monkik; Hospital by Nociconist, ID; virus transmission by mim studio, ID; Money by Icon Lauk, ID; Hospital Bed by LAFS, RU.

The complex interplay of factors leading to this increased risk is still to be fully delineated (Figure 1). However, the high prevalence of chronic comorbidities and thus risk to COVID-19 in elderly patients is thought to be key (discussed further below). Elderly patients in long-term-care facilities (LTCF) are a particularly vulnerable population and are often frail and have underlying chronic diseases, complex health needs and a requirement for medical support. Due to the COVID-19 transmission dynamics, as well as close contact between care staff and residents and a low availability of testing, there has been a rapid spread of COVID-19 within and between facilities.^8^ This has been exacerbated by a lack of personal protective equipment, absence of prompt health guidelines and the low ratio of personnel/residents during the epidemic. As a results there has been significant morbidity and mortality as a result of COVID-19 in LTCF.^8^ In England and Wales, 29.7% (15,018/50,548) deaths involving COVID-19 (registered up to 3 July 2020) were from LTCF residents.^9^ Furthermore, reports from Belgium have indicated that, as of 17 May 2020, of the 9052 fatal COVID-19 cases, 51% of those were reported to be from LTCFs.^10^

However, the burden of COVID-19 in LTCF is likely underestimated due to the lack of diagnostic testing strategies and capacity for screening.^10,11^ Particular challenges in diagnosing COVID-19 have been met with a high prevalence of asymptomatic SARS-CoV-2 positive diagnostic results. Of residents and staff in a selection of Belgium LTCFs, 73% (5695/7751) of PCR-positive cases were asymptomatic.^12^ Implementation of local and national monitoring systems for COVID-19 and other respiratory viral diseases is thus now important to identify infected patients, monitor the disease and fully understand the burden of COVID-19 in LTCFs. Identification of infected patients is also ultimately key in treatment and the prevention of SARS-CoV-2 transmission in these vulnerable patients.

### Influenza

Each year, influenza causes an estimated 1.3–2.1 million hospitalisations and associates with an average of 51,203 deaths in the US alone.^5,13^ The elderly are exposed to influenza A and B throughout their life. However, due to the vast antigenic drift and shift they do not develop immunological immunity against circulating seasonal strains. Due to their aging immune system and co-morbidities, the elderly are particularly vulnerable, with 90% of influenza-related deaths occurring in this population.^14^ Influenza infection can cause significant functional decline in older adults, and 67% and 25% have been reported to become at least temporarily housebound and bedbound, respectively.^15^ The presence of common underlying comorbidities in this population, including diabetes and respiratory and cardiovascular diseases, further increases their risk of morbidity and mortality due to influenza (Figure 1).

Influenza attack rates in LTCFs have been reported to range widely between 4% and 94%, with mortality rates during outbreaks reaching nearly 55%.^16^ Influenza-related pneumonia and cardiovascular complications occur substantially more frequently in the elderly. Furthermore, influenza infection can present as a relatively mild respiratory illness in the elderly but set off a sequence of catastrophic events that may be difficult for the clinician to link back to influenza itself.^17^ Elderly patients may present atypically without fever and simply with a cough, fatigue and confusion, thus making the true burden of influenza hard to estimate.^18^

Contrasting with seasonal influenza, the majority of symptomatic infections during pandemic influenza occur amongst young adults. This has been hypothesised to be due to prior exposure to the pandemic strain in older adults with persistent immunological memory.^19,20^ However, in the 2009 H1N1 pandemic, although fewer elderly adults developed clinical influenza, those who did were at high risk of mortality; the Californian >50-year-old population had the highest mortality rate of all age groups of between 18% and 20%.^21^ Thus, pandemic influenza is also a significant burden in the elderly.

### RSV

RSV has been estimated to cause 177,000 adult hospitalisations each year, compared with 294,000 due to influenza.^13,22^ However, healthy adults with a functioning mature immune system generally develop effective anti-RSV immunity.^23^ RSV causes significant morbidity in the immunocompromised, babies and the elderly, and is associated with 17,358 deaths in the >65-year-olds in the US each year, which increases with advancing age.^5,24^ However, geriatric physicians have little incentive to diagnose RSV infections in the elderly due to a lack of available sensitive diagnostic tests and treatments.

Prospective studies have been key in starting to understand the burden of RSV, which first became apparent in LTCFs where RSV has potential to propagate.^22^ Annual infection rates of 5–10% have been reported in LTCFs, with rates of pneumonia and mortality of 10–20% and 2–5%, respectively.^25^ However, high variability in incidence between institutions has made it hard to estimate the burden of RSV.^26^

Looking in the wider community, annual rates of hospitalisation due to RSV in healthy >50 year olds have been reported at 15.01 per 10,000 county residents.^27^ Furthermore, RSV has been reported to account for 5–15% of community acquired pneumonia and 9–10% of hospital admissions for acute cardiorespiratory diseases.^28^ A study by Falsey *et al.* found that 7.4% of elderly patients with influenza-like illness (ILI) were positive for RSV, rising to 8.7% of >75-year-olds and 12.5% of elderly patients requiring hospitalisation.^25^ Thus, the true impact of RSV is likely underestimated.

### HMPV

By the age of 25, nearly all adults have been infected with HMPV. However, infection rarely causes morbidity in young adults. HMPV reinfection does, however, account for a significant burden of morbidity in elderly adults, particularly in institutionalised elderly adults with associated frailty, immunosuppression or chronic cardiopulmonary disease.^29,30^ HMPV can cause influenza-like symptoms and lower respiratory symptoms such as wheezing and dyspnea.^31^ However, 40% of HMPV-infected elderly patients reportedly develop pneumonitis,^32^ and 2–4% of adults admitted for pneumonia are reportedly infected with HMPV.^33^ One prospective study found an annual hospitalisation rate (in all ⩾50-year-olds) due to HMPV infection of 9.82 per 10,000 residents; this compared with 11.81 for influenza.^27^ However, further studies are needed to understand the true burden of illness due to HMPV.^34^

### HRV

There are a vast number (>160) of co-circulating HRV serotypes. Therefore, despite previous HRV exposure, older adults are still susceptible to infection. Compared with influenza and RSV, HRV is usually regarded as a more benign pathogen that has comparatively low virulence and may cause asymptomatic infection or mild disease, including common cold symptoms, rhinorrhea, cough and sore throat.^35^ However, mild infection is less common in older adults, and HRV outbreaks can lead to significant morbidity and mortality.^36–38^ HRV causes particular burden in elderly patients through exacerbations in those with chronic obstructive pulmonary disease (COPD).^39–41^ HRV has been detected in 28% of >60-year-olds with upper respiratory tract infection symptoms; 26% of these were unable to perform routine household activities and nearly 20% were confined to bed. HRV has also been found in 9% of patients with radiographic evidence of pneumonia.^42^ One study looking at hospitalised pneumonia patients surprisingly found more pneumonia complications, requirement for oxygen therapy and a higher mortality rate in HRV-infected patients than in those with influenza.^43^ It is now important to fully understand the burden of all strains of HRV in the elderly, including emerging strains of HRV-C and D.^29^

### Parainfluenza and other respiratory viruses

HPIV is divided genetically and antigenically into four types, with HPIV-1 to HPIV-3 being major causes of lower respiratory infections in the elderly.^44^ Unlike most seasonal respiratory pathogens, which peak in the winter period, HPIV can infect throughout the year and cause epidemics amongst institutionalised elderly individuals.^45^ One serious HPIV3 outbreak in a LTCF with 49 elderly residents had an attack rate of 50%, with 11 radiographic pneumonias and four deaths.^46^ Although there are reports of HPIV epidemics in LTCFs, the epidemiological evidence of its impact on the wider elderly community is limited. A study using multiplex PCR in patients with ILI detected HPIV infection in 5.8 % of adults, making it the third most prevalent virus detected.^47^ However, HPIV is often not screened for in patients with acute respiratory infections and may not be reported, resulting in its true impact being underestimated.^45^

A study looking at respiratory viral infections over 3 years demonstrated that, rather than a single predominating pathogen, a wide range of respiratory viruses co-circulated in LTCFs.^48^ Further comprehensive prospective studies throughout the year in different settings using sensitive multiplex PCR for all respiratory viruses would be beneficial to try to understand the epidemiology of emerging viruses, including rotavirus, adenovirus, new coronaviruses, human bocavirus and Ki and Wu polyomaviruses.^49^ This will help to understand the epidemiology and etiological importance of each of these respiratory viruses, both individually and in the context of multiple infections. However, consideration is required about the impact of PCR confirmation of respiratory viral infection on antibiotics use by clinicians, particularly with bacterial and viral coinfection.

## Risk factors for increased morbidity and mortality in the elderly

### Atypical presentation of respiratory viral infections

Elderly patients have a high rate of complex multimorbidities and symptoms of pre-existing diseases and are more susceptible to respiratory viral infections than healthy young adults due to a multitude of factors (Figure 1). Thus, elderly individuals with a respiratory viral infection may present with respiratory distress or pneumonia. However, elderly patients may also present atypically without fever or respiratory symptoms. Often, elderly adults instead experience an exacerbation of chronic diseases, including heart failure, chronic kidney disease, COPD or diabetes.^50,51^ C-Reactive protein (CRP) and procalcitonin levels are useful biomarkers of inflammation and respiratory infections. However, difficulties have been found in diagnosing pneumonia or respiratory viral infections in the elderly.^52–55^

Reports so far have described an increased likelihood of older people with COVID-19 to have an atypical presentation.^56^ A report from France detailed presentation of the elderly with delirium, postural instability or diarrhoea as opposed to the typical presentation in younger patients.^57^ This atypical presentation may delay a COVID-19 diagnosis and increase the opportunity for SARS-CoV-2 to be transmitted, thus highlighting the importance of SARS-CoV-2 screening in elderly patients. Computed tomography has been shown to be a useful tool in diagnosing COVID-19 and other respiratory diseases in elderly patients.^58–63^ However, difficulties have been found in differentiation between viral and bacterial pneumonia.^63,64^

Elderly patients also present atypically with other respiratory viruses. A recent study by Datta *et al.* found that only 30% and 7% of hospitalised patients with influenza or RSV infection, respectively, had viral infection listed as their primary diagnosis. Instead, primary diagnoses were frequently found to be dehydration, altered mental status, falls or exacerbations of chronic diseases.^65^ Patients also presented with confusion, anorexia or a reduction in communication with the environment. This picture is further clouded by patients with obstructive lung diseases presenting with symptoms of respiratory viral infection despite not being infected.^66^

Difficulty in diagnosing respiratory viral infections in the elderly has implications for patient health as well as the swift introduction of appropriate control measures to contain respiratory viral outbreaks. A study by Gravenstein *et al.* highlighted that influenza-related morbidity has been greatly underestimated, particularly in frail older adults.^67^ Thus, the reported morbidity and mortality for respiratory viruses may represent just the tip of the iceberg.^68^ Although rapid point-of-care multiplex PCR systems for non-SARS-CoV-2 viruses are being piloting in some hospitals, such screening is usually reserved for research, clinical trials and monitoring of epidemics. The importance of accurate rapid diagnostic testing in the hospital setting is now becoming understood. However, due to the frequent atypical presentation of the elderly, accurate rapid diagnostic screening for all respiratory viruses should be routine for both symptomatic and asymptomatic elderly patients.

### Altered immune responses to viral infections

The aging immune system is becoming increasingly recognised as an important factor in driving the susceptibility of the elderly population to respiratory viral infections, with the immune system being further dysregulated in patients with underlying respiratory diseases like COPD (Figure 1).^69–73^ Immunosenescence and inflammaging are becoming increasingly understood to be driving the immune system becoming imbalanced during aging. Immunosenescence is a multifactorial aging process characterised by the gradual dysregulation and deterioration of the immune system. This impacts its capacity to function and develop long-term immune memory against respiratory viral infections.^74^ The aging immune system has been reported to have reduced B cell numbers and a dysregulated T cell compartment with thymic involution, reduced naive T cells and a higher proportion of terminally differentiated CD8 T cells in the lung.^75^ T cells have been shown to have impaired function and express exhaustion markers and lower levels of co-stimulatory molecules.^71^ A diminished humoral response in the elderly has been linked to T cell function, and waning neutralising antibodies have been found both after RSV infection and months following influenza vaccination.^76–78^

Inflammaging is a further interwoven process where the aging immune system develops elevated inflammatory mediators. This could potentially drive numerous adverse changes and disease development, including malignancies and autoimmune diseases. Furthermore, these may indirectly increase the susceptibility of the elderly to respiratory viral infections.^79^ Inflammaging is thought to influence severity of respiratory viral infections and elevated interleukin-6 (IL-6) levels have been correlated with RSV infection severity.^80,81^ This could also play a role in the predisposition of elderly patients to viral-induced cytokine storm, immunopathology and severe COVID-19 disease.^82^ New reports also highlight the potential impact of chronic antigen stimulation and persistent cytomegalovirus (CMV) reactivation in driving the aging immune system.^83^ Further work is now required to fully delineate immunosenescence and inflammaging mechanisms and how they drive susceptibility to respiratory viral infections in the elderly. This could help identify biomarkers for the most vulnerable elderly as well as immunological targets to bolster the elderly anti-viral immune system using novel therapeutics and vaccination approaches.

### Multimorbidities

Comorbidities have been found to be a key risk factor for development of severe COVID-19 disease. Docherty *et al.* demonstrated that only 22.5% of 20,133 UK patients hospitalised with COVID-19 had no comorbidity, and that 30.9% had chronic cardiac disease, 20.7% had diabetes, 17.7% had chronic pulmonary disease excluding asthma, 14.5% had asthma and 16.2% had chronic kidney disease.^84^ Thus, the high prevalence of these chronic diseases in the elderly likely plays a key role in increased COVID-19 susceptibility. The risk of heart disease on viral susceptibility is well known. The impact of viral infections on exacerbation of atrial fibrillation and heart failure has also been widely reported, as well as the increasing risk of heart attacks and stroke.^85–87^

The most vulnerable elderly adults are also reportedly 60 times more at risk of hospitalisation due to influenza infection compared with healthy adults aged 65–75.^88^ Key risk factors include very advanced age, prior hospitalisation admission for influenza infection and chronic health conditions; particularly those requiring monthly medical follow ups. Institutionalisation, immunosuppression and chronic cardiopulmonary illness are also known risk factors for severe illness due to HMPV (Figure 1).^89^ Thus, with an aging population and increased prevalence of chronic diseases, multi-morbidities may be an important indirect driver for morbidity and mortality due to respiratory viral infections.^90^ Frailty is characterised by multisystem decline, decreased physiologic reserve, impaired homeostasis, dependency and premature mortality. Frailty has been reported to have a prevalence of 7% in the community dwelling elderly and to be associated with an increased risk of influenza infection through an impaired post-influenza vaccine response.^91,92^ Infection of these vulnerable patients can thus cause significant functional impairment that can persist for months without returning to baseline or mortality. Actively screening frail patients with exacerbations of chronic disease may be important in diagnosing and treating respiratory viral infections in this vulnerable population.

Decreased mobility and changes to lung physiology, structure and function through aging may also play important roles in impacting the respiratory pump and host responses to a respiratory viral infection. Similarly, decreased functional reserve could reduce their ability to cope with increased airway resistance and decreased lung compliance as a consequence of lower-respiratory-tract infection.^93^ Malnutrition may increase the susceptibility of elderly individuals to respiratory viral infections.^94^ Undernutrition in hospitalised patients and risk of nosocomial infections have also been reported to be interrelated.^95^ Although we understand a lot about the virology of established respiratory viruses, there is still some way to go to fully understand the interplay between different factors that drive elderly susceptibility, as well as their impact on vaccine responses, including for SARS-CoV-2 (Figure 1).

### Polypharmacy

The number of elderly patients with multimorbidities is increasing and more patients are being prescribed multiple drugs. A large European study in 2707 elderly home care patients (mean age of 82 years) found that polypharmacy (defined as use of at least six medications) was identified in 51% of participants.^96^ Upon being admitted to hospital with a respiratory viral infection, these medications may be stopped or altered, potentially impacting the patient’s underlying conditions. Furthermore, morbidity due to viral infection may be accentuated by prescribed medications, including diuretics and nephrotoxic drugs, which could lead to renal failure. Immunosuppressive therapies, including steroids, cancer therapies and drugs for chronic inflammatory diseases have an array of side effects which can exacerbate chronic diseases and may also directly increase viral susceptibility as well as impacting vaccination responses.^97,98^

Polypharmacy can lead to inappropriate medications being prescribed, drug–drug interactions, an increased risk of nonadherence and adverse drug reactions (ADRs) and is a particular problem in the elderly due to their altered metabolism, reduced drug clearance and therefore greater risk for ADRs.^99^ ADRs can be multiple and non-specific, particularly in the elderly, and thus may be misinterpreted as either aging or underlying disease, resulting in further treatments, leading to a ‘prescribing cascade’.^100^ Elderly patients are also more likely to be prescribed antibiotics upon presenting with a respiratory viral infection, leading to the potential of ADRs, drug interactions and complications of underlying diseases.^101^ With the development of novel anti-viral and immunomodulatory therapeutics, the impact of polypharmacy needs further consideration.

## Prevention and treatment of respiratory viral infections

### Influenza vaccination

Influenza vaccination has been recommended as the primary prevention method for influenza infection in >65-year-olds since the 1960s,^102–105^ and not only prevents influenza infection but plays a key role in prevention of secondary events and complications, including cardiovascular morbidity and mortality.^106,107^ Vaccination of healthcare professionals (HCPs) may also be important in protecting elderly patients from viral infections and complications and has been a consideration during the COVID-19 pandemic. However, the true impact on both influenza and SARS-CoV-2-related infection is yet to be demonstrated conclusively.^108^

Vaccination has been hugely successful in reducing morbidity and mortality associated with influenza infection in the general population. However, there is limited data about comparative vaccine effectiveness in >65-year-olds (Table 1).^109^ Serological studies have found lower and waning humoral responses in older adults. Therefore, clinical protection is not likely to persist throughout the whole year, highlighting the requirement for more immunogenic vaccines.^110^ A new high-dose trivalent inactivated influenza vaccine that contains four times the amount of haemagglutinin has been produced and has been licenced for use in the US since 2009. This has been shown to produce higher antibody responses and be more effective in adults >65 years in preventing overall mortality. However, in a recent meta-analysis, protection with the high-dose trivalent vaccine was found to be only 22.2% [95% confidence interval (CI): –18.2 to 48.8%] against post-influenza mortality.^111^ A quadrivalent flu vaccine has also been licenced in the US since 2013; this encompasses an additional B-like virus strain and has been shown to provide similar immunogenicity induced by the strains in the trivalent vaccine.^112,113^ Current influenza vaccines need to be administered annually to cover the circulating strains. Further work for effective vaccines in the elderly is essential in both influenza and other respiratory viruses including RSV and SARS-CoV-2.

**Table 1.** Efficacy of influenza vaccinations in the elderly.

	Study (vaccine)	Population (number of participants)	Flu season(s)	Outcome/Comparison	Result	Reference
1	Seasonal flu vaccine	>65 years (3402) 18–50 years: (1131)	2011–2016	Vaccine effectiveness >65 years *versus* 18–49 years	H3N2: 14% *versus* 21% H1N1: 49% *versus* 48% Influenza B: 62% 55%	Russell *et al.*^114^
2	Seasonal flu vaccine	>65 years: (4643) 18–58 years: 1151	1986–2002	Vaccine effectiveness >65 years *versus* 18–58 years	H3N2: 74% *versus* 84% H1N1 69% *versus* 83% Influenza B: 78% *versus* 67%	Goodwin *et al.*^115^
3	QIV *versus* TIV	>61 years: (989) 18–60 years: (991)	2015–2016	Seroconversion rates >61 *versus* 18–60 years	⩾73.3% *versus* ⩾91.6%^a^	van de Witte *et al.*^113^
4	TIV-HD *versus* TIV	>65 years TIV-HD: (6117) >65 years TIV: (3055)	2009–2010	Relative efficacy >65 years TIV-HD *versus* >65 years TIV	12.6%	DiazGranados *et al.*^116^
5	TIV-HD *versus* TIV	>65 years TIV-HD: (15,991) >65 years TIV: (15,998)	2011–2012	Relative efficacy >65 years TIV-HD *versus* >65 years TIV	24.2%	DiazGranados *et al.*^117^
6	ATIV *versus* TIV	>65 years ATIV: (165) >65 years TIV: (62)	2011–2012	Vaccine effectiveness >65 years ATIV *versus* >65 TIV	58% (*p* < 0.04) *versus* –7% (0.97)	Van Buynder *et al.*^118^

a Authors reported a non-inferior immunogenicity of QIV *versus* TIV to the matched influenza strains.

ATIV, adjuvanted trivalent inactivated vaccine; HD, high dose; TIV, trivalent inactivated vaccine; QIV, quadrivalent inactivated vaccine.

### Other vaccines and novel vaccination approaches

Vaccination is essential for the prevention of respiratory viral infection and provides not only protection to the individual but herd immunity to others; this is particularly important in elderly patients either in hospital or a LTCF. COVID-19 vaccines are currently under development and promising results have been demonstrated in providing 94% protection in the over-65-year-olds.^119,120^ However, widespread availability is still some time away and the ability to induce lasting protection in elderly patients is still to be demonstrated.

Although vaccinations exist for influenza there are still no vaccines available for other respiratory viruses such as RSV.^121^ However, substantial progress has been made in understanding the burden and immunobiology of RSV, and there are now over 60 vaccine candidates currently in preclinical and clinical trials using various platforms.^122^ Vaccines for HRV have been problematic due to the limited antigenic cross-reactivity between the >160 serotypes of HRV, and it seems unlikely that protection against all serotypes of HRV will be achieved with a single immunogen. New pre-clinical studies have, however, used peptide immunogens successfully to generate cross-reactive antibodies. Furthermore, T cell inducing strategies are also now being explored to generate a broadly cross-reactive vaccine.^123^ New innovations are under development for the advent of vaccines for HRV and other respiratory viruses such as HMPV. Supplementing these with novel vaccines against key bacterial pathogens that exacerbate lung diseases will be key in caring for the vulnerable older population.^124^

Early strategies to improve the immune response in elderly individuals following vaccination included increasing the antigen dose or co-administration with an adjuvant. An influenza-adjuvant vaccine using MF59 (an oil-in-water emulsion of squalene oil) has been approved for use in the US since 2015. However, randomized controlled trials comparing the efficacy of high dose and MF59-adjuvanted influenza vaccines have not as yet been undertaken.^125^ New liposome-based immunostimulant-containing adjuvants may also have potential in improving vaccination efficacy. For example, ASO1 has been approved recently for use in older adults following its efficacy against herpes zoster in clinical trials, and may have broader potential for vaccination against respiratory viruses.

Various promising toll-like receptor (TLR) stimulants are also in development. A synthetic variant of LPS from Salmonella Minnesota acts as a TLR4 agonist. This has been tested in a phase I trial with a RSV F-protein vaccine candidate.^126^ Other TLR agonists include imiquimod, a TLR7/8 agonist that has been shown to enhance efficacy of the trivalent influenza vaccine in young adults when applied topically.^127^

There is an increasing understanding that the site of vaccine delivery can influence the resultant immune response and vaccine efficacy.^128^ Advancements in less invasive delivery systems mean delivery of vaccines in the lungs could be feasible in the future. However, with the challenges of vaccinations in the elderly, other novel approaches for vaccine development in this population are being developed. Senolytic and other immunomodulatory drugs may have potential in enhancing vaccine responses and are starting to be translated into clinical trials.^129^ For example the mTOR inhibitor RAD001 has been shown to enhance vaccine responses in the elderly in a phase IIa trial, potentially through altering cellular metabolism and increasing expression of interferon (IFN)-related genes.^130^

With the ever-increasing potential for integrating different omics datasets, systems vaccinology is now being applied widely to understand immunological responses to vaccinations in different populations. Using this approach, and incorporating immunobiography and clinical, microbiome, immunome and other omic data, there is real promise for the development of novel vaccines and adjuvants. Furthermore, there is potential to stratify elderly subpopulations for personalised vaccination approaches in the future using this approach.^121,129,131,132^

### Antiviral therapeutics

The investigation into potential COVID-19 therapeutics is still ongoing, with a variety of novel therapeutics being trialled.^133–138^ However, exogenous IFN-β has potential in preventing respiratory viral infections in the elderly and early results from a randomised controlled trial demonstrate the efficacy of IFN-β in improving symptoms in hospitalised patients with COVID-19.^133,139^ Dexamethasone has also been shown to be effective at decreasing mortality in up to a third of hospitalised patients with severe respiratory COVID-19 complications and is now being widely used for COVID-19 patients with a requirement for oxygen.^140^ However, the relative efficacy of these emerging treatments in elderly patients is still to be delineated.

The mainstay of treating influenza is initiation of anti-viral medication as soon as possible.^141^ A recent phase III trial demonstrated that baloxavir was effective at reducing high risk influenza complications and decreasing the time to improvement of influenza symptoms.^142^ Clinical trials have shown efficacy of anti-viral medications when given to patients with uncomplicated influenza infections within 48 h.^143^ However, there have been no completed randomised, placebo-controlled trials in hospitalised patients. Given the frequency of complicated influenza infections and late diagnosis of influenza in the elderly, the efficacy of anti-viral medications in the elderly needs further evaluation. Antivirals such as Oseltamivir are also known to have various side effects and new drug interactions are coming to light, including an interaction with warfarin.^143^ Particular consideration should, therefore, be given prior to treatment of vulnerable elderly patients and influenza infection should be confirmed with sensitive diagnostics. Novel antiviral compounds are also being investigated for treatment of influenza;^144^ these include the development of monoclonal and polyclonal influenza-targeting antibodies, convalescent plasma and small-molecule polymerase inhibitors.

As with influenza infection, management of elderly patients with other acute respiratory viruses is predominantly through supportive care. This is an active process with frequent monitoring as well as hydration, as required supplemental oxygen, treatment of fever and use of bronchodilators until the patient recovers.^145^ Antihistamines and nonsteroidal anti-inflammatory drugs may relieve some symptoms of viral infection. However, these are often contraindicated in the oldest patients because of underlying kidney disease, risk of gastrointestinal (GI) bleeding and confusion.^146^ No antiviral drugs that shorten the duration of HRV illness are currently approved for clinical use.^147^

Although there are promising emerging candidates for the treatment of RSV infection in the elderly, there are also still no effective antiviral treatments on the market. Various novel antiviral strategies are currently being investigated and have previously been reviewed.^148^ These include targets to prevent fusion of RSV with epithelial cells such as antibodies targeting the RSV fusion protein (including Palivizumab), which have been licenced for prophylactic use in high-risk children. Drugs inhibiting viral replication such as nucleoside analogues (including JNJ-64041575) are also being investigated, and phase II clinical trials have demonstrated their antiviral effects and efficacy in reducing clinical symptoms. Small interfering RNAs have been trialled to downregulate viral protein production. A clinical trial in lung transplant patients used a small interfering (si)RNA (ALN-RSV01), which targets the RSV nucleocapsid protein. This showed that, although it did not show antiviral effects, it did improve the daily total symptom score.^149^

Immunomodulatory anti-viral agents are currently also under investigation to bolster the immune system for treatment of respiratory viruses. These include a COX-2 inhibitor (celecoxib), statins, pioglitazone and azithromycin.^150^ Recombinant versions of natural innate immune proteins surfactant proteins A and D may also have potential for development into antiviral therapeutics and have key roles as both broadly selective innate immune molecules and immunomodulators.^151–158^

## Conclusion

COVID-19 has already caused >2.3 million deaths and has highlighted the impact and global burden of respiratory viral infections, particularly in the elderly. Although influenza is also a key respiratory pathogen, the importance of other respiratory viruses is starting to become apparent. Compared with young adults, respiratory viral infection in >65-year-olds is associated with increased morbidity and mortality. Elderly patients may present with severe lower respiratory tract infections, pneumonia or exacerbation of chronic multimorbidities. Further challenges are faced due to elderly patients often presenting without fever or respiratory symptoms but with atypical symptoms including weakness, confusion and falls. This leads to a failure in diagnosis, increased morbidity, prescription of additional medications and further nosocomial spread. It is now essential to introduce sensitive, rapid point-of-care diagnostics to identify all viruses and treat infected elderly patients. This will facilitate an understanding of the true burden of respiratory viruses. To protect the aging immune system, it is now key to couple this with the development of augmented vaccination strategies and novel anti-viral therapeutics.

## Supplemental Material

sj-pdf-1-tar-10.1177_1753466621995050 – Supplemental material for Respiratory viral infections in the elderly Click here for additional data file. Supplemental material, sj-pdf-1-tar-10.1177_1753466621995050 for Respiratory viral infections in the elderly by Alastair Watson and Tom M. A. Wilkinson in Therapeutic Advances in Respiratory Disease

sj-pdf-2-tar-10.1177_1753466621995050 – Supplemental material for Respiratory viral infections in the elderly Click here for additional data file. Supplemental material, sj-pdf-2-tar-10.1177_1753466621995050 for Respiratory viral infections in the elderly by Alastair Watson and Tom M. A. Wilkinson in Therapeutic Advances in Respiratory Disease

sj-pdf-3-tar-10.1177_1753466621995050 – Supplemental material for Respiratory viral infections in the elderly Click here for additional data file. Supplemental material, sj-pdf-3-tar-10.1177_1753466621995050 for Respiratory viral infections in the elderly by Alastair Watson and Tom M. A. Wilkinson in Therapeutic Advances in Respiratory Disease
