# Supplementary material for: Respiratory viral infections in the elderly
Source: Ther Adv Respir Dis. 2021 Mar 21;15:1753466621995050. doi: 10.1177/1753466621995050 (PMC7989115; doi:10.1177/1753466621995050)
Supplement: sj-pdf-2-tar-10.1177_1753466621995050 – Supplemental material for Respiratory viral infections in the elderly [file sj-pdf-2-tar-10.1177_1753466621995050.pdf]

Reviewer 1 v.1

Comments to the Author

Thank you for allowing me to review your paper entitled "Respiratory Viral Infections in the Elderly." This paper provides a slightly superficial but complete review of the major issues of RVI in the elderly. The paper is long and could be shortened in some areas (i.e. discussion of drugs that were developed a decade or more ago that aren't going anywhere so not worth being in the text) and other areas (limited data on baloxavir, limited data on various vaccination approaches in elderly including more data than immunogenicity of high dose).
