# Supplementary material for: Respiratory viral infections in the elderly
Source: Ther Adv Respir Dis. 2021 Mar 21;15:1753466621995050. doi: 10.1177/1753466621995050 (PMC7989115; doi:10.1177/1753466621995050)
Supplement: sj-pdf-3-tar-10.1177_1753466621995050 – Supplemental material for Respiratory viral infections in the elderly [file sj-pdf-3-tar-10.1177_1753466621995050.pdf]

Reviewer 2 v.1

Comments to the Author

Major

This is a timely review article concerning respiratory viral infections in the elderly. Since there has been a global COVID-19 pandemic which has caused over 600,000 deaths so far, and increased age has been shown to be the greater risk for morbidity and mortality in the population. Thus the burden of respiratory viral infections including SARS-Cov2 in the elderly is becoming an increasing unmet clinical need. Their text is well described and references are identical. However, no figures and tables are available.

There is no cardinal attitude for the readers of the Journal a various data and plenty of viral infection data should be presented as figures and tables.
