# Supplementary material for: Respiratory viral infections in the elderly
Source: Ther Adv Respir Dis. 2021 Mar 21;15:1753466621995050. doi: 10.1177/1753466621995050 (PMC7989115; doi:10.1177/1753466621995050)
Supplement: sj-pdf-1-tar-10.1177_1753466621995050 – Supplemental material for Respiratory viral infections in the elderly [file sj-pdf-1-tar-10.1177_1753466621995050.pdf]

Author Response

Dear Editor and Reviewers,

We thank the reviewers for their insightful comments which we have compiled and used to update the manuscript accordingly, we believe that this manuscript is now improved as a result. We have written an expanded response to each of these comments in detail below.

Reviewer 1

Reviewer: Thank you for allowing me to review your paper entitled "Respiratory Viral Infections in the Elderly." This paper provides a slightly superficial but complete review of the major issues of RVI in the elderly. The paper is long and could be shortened in some areas (i.e. discussion of drugs that were developed a decade or more ago that aren't going anywhere so not worth being in the text) and other areas (limited data on baloxavir, limited data on various vaccination approaches in elderly including more data than immunogenicity of high dose).

Author: We thank the reviewer for these suggestions and in response have now gone through the manuscript and shortened and edited throughout. In line with these comments, we have condensed the section on antiviral drugs through removal of discussion around old drugs which have not been shown to be effective in the elderly including Ribavirin, neuraminidase inhibitors, M2 inhibitors and cap-dependent endonuclease inhibitors. This section now focuses on only the relevant current and future potential treatments for modulating viral infection in the elderly. In specific response to the reviewer’s suggestion, we have added the recent work on Baloxavir by Ison et al. 2020 published in The Lancet Infectious Diseases which demonstrates the efficacy of early therapy with Baloxavir for patients at high risk of complications of influenza, with potential to demonstrate efficacy in elderly populations in future clinical trials. Furthermore, we have now expanded on novel vaccination approaches for the elderly throughout the “Vaccines and Novel Vaccination Approaches”, in addition we have specifically added 2 additional paragraphs to address this useful comment (Lines 382-397 within the clean manuscript).

Reviewer 2

Reviewer: Major

This is a timely review article concerning respiratory viral infections in the elderly. Since there has been a global COVID-19 pandemic which has caused over 600,000 deaths so far, and increased age has been shown to be the greater risk for morbidity and mortality in the population. Thus, the burden of respiratory viral infections including SARS-Cov2 in the elderly is becoming an increasing unmet clinical need. Their text is well described and references are identical. However, no figures and tables are available. There is no cardinal attitude for the readers of the Journal a various data and plenty of viral infection data should be presented as figures and tables.

Author: We thank the reviewer for their suggestions and appreciate these useful comments. In line with these suggestions, we have now added a table to summarise key clinical trials reporting the efficacy of influenza vaccinations in the elderly. We have additionally added a figure encompassing the important factors driving the increased susceptibility of the elderly to respiratory viral infections,

which we feel acts as a cardinal figure for the narrative of this review. We thank the reviewer for highlighting the need to add these components to the manuscript, and we hope that they now agree that this manuscript is suitable for publication.
